# Disseminated *Nocardia* infection with a lesion occupying the intracranial space complicated with coma: a case report

**DOI:** 10.1186/s12879-020-05569-4

**Published:** 2020-11-17

**Authors:** Mei-Hong Yu, Xiao-Xin Wu, Chun-Lei Chen, Song-Jia Tang, Jian-Di Jin, Cheng-Li Zhong, Jing Fu, Jie-Qin Shi, Lan-Juan Li

**Affiliations:** 1 State Key Laboratory for Diagnosis and Treatment of Infectious Diseases, Collaborative Innovation Center for Diagnosis and Treatment of Infectious Diseases, National Clinical Research Center for Infectious Diseases, The First Affiliated Hospital, Zhejiang University School of Medicine, 79 Qingchun Road, Hangzhou, 310003 Zhejiang China; 2 Department of Plastic Surgery, Affiliated Hangzhou First People’s Hospital, Zhejiang University School of Medicine, 261 Huansha Road, Hangzhou, 310000 Zhejiang China

**Keywords:** *Nocardia cyriacigeorgica*, Intracranial occupying lesion, Antibiotic therapy, Surgical drainage

## Abstract

**Background:**

Disseminated *Nocardia* infection is a disease that is easily overlooked in patients with lesions occupying the intracranial space complicated with coma. Early diagnosis and treatment are crucial.

**Case presentation:**

A 65-year-old man was admitted to the First Affiliated Hospital of Zhejiang University in October 2018 with weakness in the right limbs for 3 days and altered consciousness for 1 day. Five months earlier, he had been diagnosed with membranous kidney disease and had received cyclophosphamide and prednisone. At admission, the white blood cell count was 1.37 × 10^10^/L (with 86.4% neutrophils), and C-reactive protein was 115.60 mg/L. Imaging examinations revealed a lesion occupying the intracranial space, lung infection, and multiple abscesses in the rhomboid muscle. The abscesses were drained. Pus culture confirmed *Nocardia cyriacigeorgica* infection. With antibiotics and vacuum-sealed drainage of the back wound, the patient improved and was discharged from the hospital.

**Conclusions:**

This case report shows that infection should be considered during the differential diagnosis of lesions in the intracranial space, especially in patients receiving immunosuppressive treatment. In patients with disseminated *N. cyriacigeorgica* infection, combination antibiotic therapy and surgical drainage of localised abscesses can be effective.

## Background

Nocardiosis is an acute, subacute, or chronic infectious disease that may be localised or disseminated; it is characterised by suppurative or granulomatous inflammation. It is usually diagnosed in adults aged 30–50 years, with male predominance, and mostly affects individuals with severe immune dysfunction [1, 2]. Patients mostly present with non-specific features such as fever, cough with expectoration, chest pain, fatigue, poor appetite, high white blood cell and neutrophil counts, and elevated blood inflammatory indices (C-reactive protein, calmodulin). The diagnosis is therefore easily missed [3]. Confirmation of diagnosis requires isolation of *Nocardia* bacteria from blood, sputum, pus, drainage, tissue, or cerebrospinal fluid specimens. We report a rare case of disseminated *Nocardia cyriacigeorgica* infection in an immunosuppressed man that was successfully managed with a combination of antibiotic therapy and surgery.

## Case presentation

**Fig. 1:**
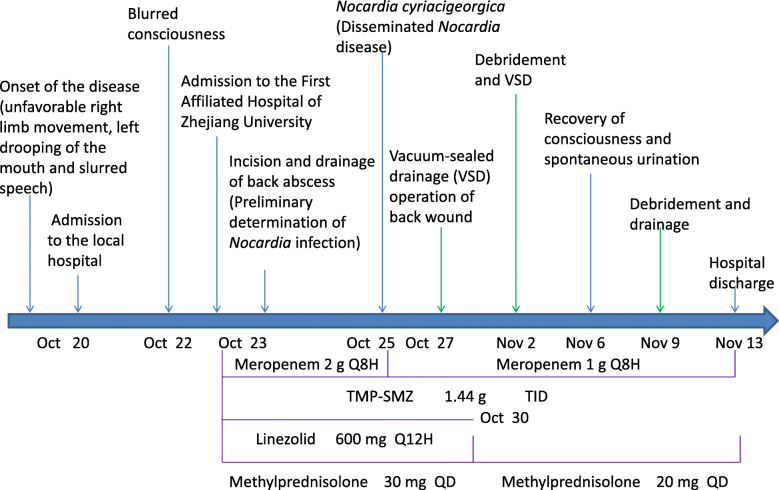
Treatment flow diagram. TMP-SMZ, trimethoprim–sulfamethoxazole

**Fig. 2:**
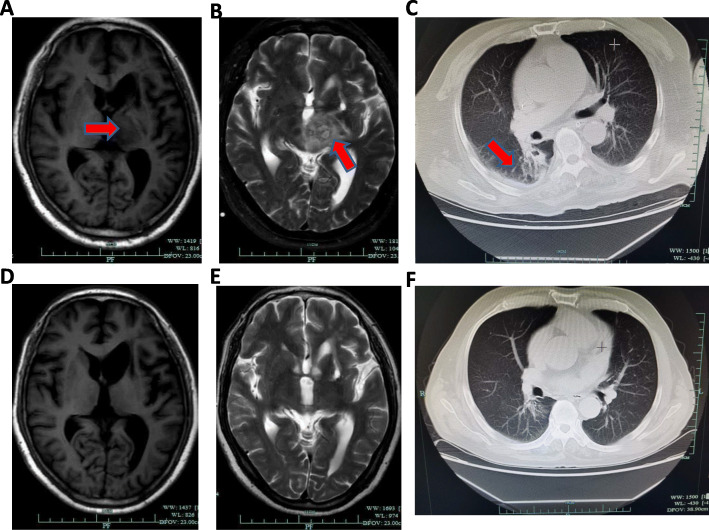
Lung and brain images of the patient at admission and at the 6-month follow-up. **a**: T1 image of brain at admission (red arrow indicates abscess). **b**: T2 image of brain at admission (red arrow indicates abscess). **c**: Cross-sectional image of the chest at admission showing the area of infection in the lung (red arrow). **d**: T1 image of brain at the 6-month follow-up. **e**: T2 image of brain at the 6-month follow-up. **f**: Cross-sectional image of chest at the 6-month follow-up

A 65-year-old man was admitted to the First Affiliated Hospital of Zhejiang University in October 2018, with weakness in the right limbs for 3 days and altered consciousness for 1 day (Fig. 1). Three days earlier, he developed sudden weakness and numbness in the right limbs, accompanied by slurred speech and deviation of the mouth to the left. At that time, he had no disturbance of consciousness, jaundice, headache, or dizziness. Computed tomography (CT) at the local hospital showed a circular low-density area in the left thalamus and midbrain. Acute cerebral infarction was suggested, and the patient was started on antiplatelet drugs. However, his condition worsened over the next 2 days and he developed fever (maximum body temperature, 38.7 °C) and altered consciousness. Skull magnetic resonance imaging (MRI) showed a lesion occupying the intracranial space (Fig. 2), and the patient was transferred to our hospital for further management.

The patient had been diagnosed with membranous kidney disease 5 months earlier and had received cyclophosphamide (cumulative dose, 1.8 g) with prednisone (48 mg once daily for 4 months, followed by 32 mg once daily for 1 month). A month earlier, he had been hospitalised in a local hospital for treatment of a lung infection. In addition, he complained of swelling and pain in his left upper back and neck 5 days prior, and B-mode ultrasonography at a local hospital revealed multiple abscesses in the left rhomboid muscle.

The patient had cough and expectoration. The phlegm was white mucilaginous sputum. On auscultation, laboured breathing in both the lungs, with no dry or wet rales, was observed. Upon admission to our hospital, his blood examination findings were as follows: white blood cell count, 1.37 × 10^10^/L (with 86.4% neutrophils); serum creatinine, 45 μmol/L; urea, 5.77 mmol/L; hypersensitive C-reactive protein, 115.60 mg/L; procalcitonin, 0.25 ng/mL; serum albumin, 30.4 g/L; total bilirubin, 23.9 μmol/L; indirect bilirubin, 17.0 μmol/L; serum glutamic pyruvic transaminase, 99 U/L; serum glutamic oxaloacetic transaminase, 142 U/L; and lactic dehydrogenase, 371 U/L. The cerebrospinal fluid appeared normal.

**Fig. 3:**
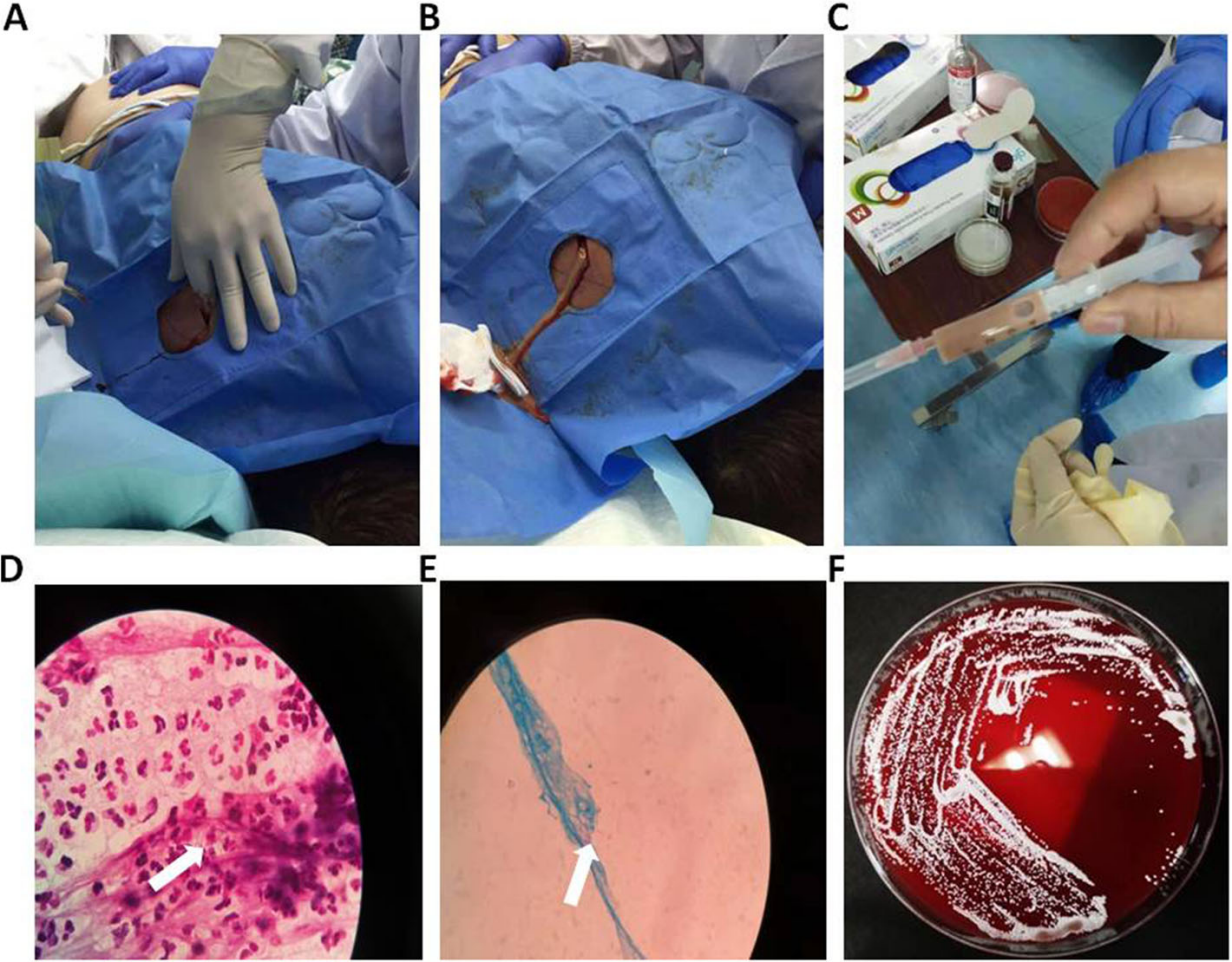
Treatment of the back abscess. **a**: Incision of back abscess; **b**: drainage of back abscess; **c**: appearance of the aspirated fluid. **d**: Gram’s stain of the aspirated pus showing a large number of white blood cells along with a small number of gram-positive bacteria (white arrow). **e**: Weak acid-fast staining (white arrow). **f**: Growth of a large number of colonies on the medium

Soft fluctuant swellings, 5 cm in diameter with an unclear boundary, were present on his right back, left shoulder, and back. The overlying and surrounding skin were red. We found multiple abscesses in the muscle layer on B-mode ultrasonography. The clinical and ultrasonographic findings were suggestive of multiple abscesses. Under local anaesthesia, a needle was inserted into the abscess cavity on the back, and greyish-white purulent fluid was aspirated. Microbiological examination of the fluid revealed a small number of gram-positive acid-fast bacterial cells. Samples were incubated at 35 °C on Columbia Blood Agar for 3 days. *N. cyriacigeorgica* was identified using matrix-assisted laser desorption/ionisation time-of-flight mass spectrometry (MALDI-TOF MS; Microflex, Bruker, Billerica, MA). The clinical and microbiological findings were suggestive of disseminated nocardiosis (Fig. 3).

Treatment was started with trimethoprim–sulfamethoxazole, along with linezolid, methylprednisolone, and meropenem (Fig. 1). Four days after the start of treatment, the blood examination results were as follows: white blood cell count, 1.10 × 10^10^/L (83.3% neutrophils); serum albumin, 32.8 g/L; serum glutamic pyruvic transaminase, 119 U/L; serum glutamic oxaloacetic transaminase, 43 U/L; and hypersensitive C-reactive protein, 8.22 mg/L.

Vacuum-sealed drainage and chronic ulcer repair were also performed for the abscess on the back. Two weeks after admission, the patient had recovered full consciousness. He was discharged from the hospital on 13 November 2018 after confirming that the C-reactive protein level had returned to normal. He was advised to continue trimethoprim–sulfamethoxazole (3 tablets [1.44 g] 3 times daily) for a total of 12 months. At the 6-month follow-up after discharge, imaging examinations showed absorption of the brain abscess and improvement of lung inflammation (Fig. 2). The patient is currently living independently.

## Discussion and conclusions

*Nocardia* is a genus of gram-positive aerobic bacteria belonging to the order Actinomycetes. *Nocardia* is widely distributed in soil and water but is not part of the normal human flora [4]. To date, 92 strains have been found in the genus *Nocardia*, among which the main pathogens are *N. asteroides*, *N. brasiliensis*, and *N. farcinica* [5, 6].The infection is usually confined to the lungs, followed by the skin and other sites. Infection of the brain is relatively rare [7]. Patients with immunodeficiencies are more likely to develop nocardiosis [7]. In a multicentre study in China, *N. cyriacigeorgica* was the second most common species of *Nocardia* and no cases of intracranial infection were identified [8]. Although intracranial infections caused by *N. cyriacigeorgica* are rare, they are still reported in patients with human immunodeficiency virus infection or diabetes [8, 9].

Antibiotics reported to be effective against *Nocardia* include sulphonamides, aminoglycosides, carbapenems, quinolones, and tetracycline. High-dose, long-course trimethoprim–sulfamethoxazole is the first choice. However, resistance to sulphonamides is being increasingly reported; thus, it should ideally be provided in combination with two or more different kinds of antibiotics. In vitro drug sensitivity tests show that the multi-drug resistant *Nocardia* is sensitive to linezolid and is therefore recommended for treatment of patients with severe or disseminated disease, allergy to sulphonamides, or poor response to other drugs [10, 11]. Meropenem was also recommended as the disease has a tendency to progress. Meanwhile, the dose and duration of antibiotic treatment depend on the site of infection and the patient’s immune status. For patients with severe disseminated infection involving the central nervous system, combination treatment should be considered [12]. Three-drug antimicrobial therapy (meropenem, linezolid, and trimethoprim–sulfamethoxazole) was suggested for patients with disseminated *Nocardia* infection (including lesions occupying the intracranial space) [13]. Prognosis is related to the severity of the underlying disease, the site of infection, the patient’s immune function, the presence of drug resistance, and the timeliness of institution of treatment.

Herein, we report a case of disseminated nocardiosis in China caused by *N. cyriacigeorgica*. This patient was successfully managed with a combination of antibiotic therapy and surgical drainage. The prognosis of nocardiosis is good with comprehensive treatment. We have summarised several points of experience. First, it is very important to conduct a careful physical examination in the clinic. Second, back abscesses should be punctured and drained in time with a bacterial smear and culture performed. Early diagnosis is crucial. Third, a strong combination of antimicrobial therapy and surgical drainage is very important for treating disseminated *Nocardia* infection. Finally, *Nocardia* infection should be considered during the differential diagnosis of a lesion occupying the intracranial space, especially in an immunosuppressed patient.

## Data Availability

All data and materials are available in the manuscript.

## Abbreviations

CT: Computed tomography
MRI: Magnetic resonance imaging
TMP-SMZ: Trimethoprim–sulfamethoxazole
